# APE1/Ref‐1 knockdown in pancreatic ductal adenocarcinoma – characterizing gene expression changes and identifying novel pathways using single‐cell RNA sequencing

**DOI:** 10.1002/1878-0261.12138

**Published:** 2017-10-19

**Authors:** Fenil Shah, Emery Goossens, Nadia M. Atallah, Michelle Grimard, Mark R. Kelley, Melissa L. Fishel

**Affiliations:** ^1^ Department of Pediatrics Herman B Wells Center for Pediatric Research Indiana University School of Medicine Indianapolis IN USA; ^2^ Department of Statistics Purdue University West Lafayette IN USA; ^3^ Purdue University Center for Cancer Research Purdue University West Lafayette IN USA; ^4^ Department of Pharmacology & Toxicology Indiana University School of Medicine Indianapolis IN USA; ^5^ Department of Biochemistry and Molecular Biology Indiana University School of Medicine Indianapolis IN USA

**Keywords:** APE1, pancreatic ductal adenocarcinoma, Ref‐1, RNA sequencing, single cell

## Abstract

Apurinic/apyrimidinic endonuclease 1/redox factor‐1 (APE1/Ref‐1 or APE1) is a multifunctional protein that regulates numerous transcription factors associated with cancer‐related pathways. Because APE1 is essential for cell viability, generation of APE1‐knockout cell lines and determining a comprehensive list of genes regulated by APE1 has not been possible. To circumvent this challenge, we utilized single‐cell RNA sequencing to identify differentially expressed genes (DEGs) in relation to APE1 protein levels within the cell. Using a straightforward yet novel statistical design, we identified 2837 genes whose expression is significantly changed following APE1 knockdown. Using this gene expression profile, we identified multiple new pathways not previously linked to APE1, including the EIF2 signaling and mechanistic target of Rapamycin pathways and a number of mitochondrial‐related pathways. We demonstrate that APE1 has an effect on modifying gene expression up to a threshold of APE1 expression, demonstrating that it is not necessary to completely knockout APE1 in cells to accurately study APE1 function. We validated the findings using a selection of the DEGs along with siRNA knockdown and qRT‐PCR. Testing additional patient‐derived pancreatic cancer cells reveals particular genes (*ITGA1*,*TNFAIP2*,*COMMD7*,*RAB3D*) that respond to APE1 knockdown similarly across all the cell lines. Furthermore, we verified that the redox function of APE1 was responsible for driving gene expression of mitochondrial genes such as *PRDX5* and genes that are important for proliferation such as *SIPA1* and *RAB3D* by treating with APE1 redox‐specific inhibitor, APX3330. Our study identifies several novel genes and pathways affected by APE1, as well as tumor subtype specificity. These findings will allow for hypothesis‐driven approaches to generate combination therapies using, for example, APE1 inhibitor APX3330 with other approved FDA drugs in an innovative manner for pancreatic and other cancer treatments.

**Abbreviations**
ANCOVA: analysis of covariance
APE1/Ref‐1: apurinic/apyrimidinic endonuclease 1/redox factor‐1
BCRP: breast cancer resistance protein
COMMD7: copper metabolism domain‐containing protein 7
DEGs: differentially expressed genes
EIF2: eukaryotic initiation factor 2
GEO: Gene Expression Omnibus
GO: Gene Ontology
HIF1α: hypoxia‐inducible factor 1α
IPA: ingenuity pathway analysis
ITGA1: integrin subunit alpha 1
PDAC: pancreatic ductal adenocarcinoma
PRDX5: peroxiredoxin 5
qRT‐PCR: quantitative real‐time PCR
scRNA‐seq: single‐cell RNA sequencing
SIPA1: signal‐induced proliferation‐associated 1
siRNA: small interfering RNA
TNFAIP2: tumor necrosis factor alpha‐induced protein 2

## 1 Introduction

Apurinic/apyrimidinic endonuclease 1/redox factor‐1 (APE1/Ref‐1; henceforth referred to as APE1) is a multifunctional protein that is involved in repairing DNA damage via its endonuclease activity in base excision repair (Fung and Demple, 2005; Izumi *et al*., 2005; Jiang *et al*., 2009; Kelley *et al*., 2014), and using its redox protein–protein signaling function to control the activity of numerous transcription factors such as STAT3, NFκB, AP‐1, p53, and hypoxia‐inducible factor 1α (HIF1α), among others (Cardoso *et al*., 2012; Fishel *et al*., 2015; Gaiddon *et al*., 1999; Jiang *et al*., 2010; Kelley *et al*., 2012; Lando *et al*., 2000; Logsdon *et al*., 2016). It also contributes to the removal of damaged bases within RNA (Poletto *et al*., 2016; Vascotto *et al*., 2014).

APE1 expression is increased in several cancers such as pancreatic (Jiang *et al*., 2010), prostate (Kelley *et al*., 2001), cervical (Xu *et al*., 1997), gliomas (Bobola *et al*., 2004), lung (Yoo *et al*., 2008), bladder (Shin *et al*., 2015), colon (Lou *et al*., 2014), and ovarian cancers (Al‐Attar *et al*., 2010; Zhang *et al*., 2009), and this increase is associated with resistance to radiation and chemotherapy, leading to poorer patient prognosis (Sharbeen *et al*., 2015). Based on its involvement in cancer, and its regulation of several transcription factors associated with cancer‐related pathways, APE1 has become a prime target for anticancer therapies (Fishel and Kelley, 2007; Kelley *et al*., 2014). Particular interest has been assigned to determining the different genes and pathways affected by APE1.

While a number of studies have investigated genes regulated by APE1, and specifically its redox signaling function (Cardoso *et al*., 2012; Fishel *et al*., 2015; Gaiddon *et al*., 1999; Jiang *et al*., 2010; Kelley *et al*., 2012; Lando *et al*., 2000; Logsdon *et al*., 2016; Nishi *et al*., 2002; Xanthoudakis *et al*., 1992), it has been difficult to compile a comprehensive list of genes regulated by APE1 as it is essential for cell viability. APE1‐knockout in mice results in embryonic lethality, postimplantation, between days E5‐E9 (Ludwig *et al*., 1998; Xanthoudakis *et al*., 1996). Subsequently, it is not possible to generate stable APE1‐knockout cell lines (Tell *et al*., 2009). Approaches to circumvent this dilemma have utilized conditional knockouts and siRNA knockdowns (Fung and Demple, 2005; Izumi *et al*., 2005; Jiang *et al*., 2010). For example, using siRNA knockdowns, our laboratory has previously identified APE1 directly regulating STAT3 transcriptional activity (Cardoso *et al*., 2012), suppressing Nrf2‐induced gene expression (Fishel *et al*., 2015) and, most recently, regulating carbonic anhydrase 9 (CA9) via HIF‐1α under hypoxic conditions (Logsdon *et al*., 2016).

While APE1 knockdowns via siRNA are useful, this approach produces a heterogeneous population, resulting in cells with differing amounts of the APE1 protein. Additionally, siRNA knockdowns are transient with APE1 expression recovering over time (Jiang *et al*., 2010). Consequently, there may be a limit to the amount of information gained using APE1 siRNA in a mixed population. In order to address this problem and more accurately detect changes to the potential numerous effectors regulated by APE1, we utilized single‐cell RNA sequencing (scRNA‐seq).

In the studies presented here, APE1 was knocked down using siRNA in low‐passage patient‐derived pancreatic ductal adenocarcinoma (PDAC) cells, and the resulting cells, together with control cells treated with scrambled siRNA, were analyzed using scRNA‐seq. The data were analyzed by employing a straightforward yet novel statistical design that utilized the BPSC r package (Vu *et al*., 2016) to accurately determine which genes were differentially expressed in response to varying APE1 expression levels per cell. Pathway analysis identified numerous pathways influenced by APE1 knockdown, including eukaryotic initiation factor 2 (EIF2) signaling, protein kinase A signaling, and mechanistic target of Rapamycin (mTOR) signaling. Using data from The Cancer Genome Atlas (TCGA) (Cancer Genome Atlas Research Network *et al*., 2013), the clinical relevance of the differentially expressed genes (DEGs) was assessed by fitting a Cox proportional hazards model. A number of the DEGs were validated in a heterogeneous APE1 population using siRNA knockdown and quantitative real‐time PCR (qRT‐PCR). A subgroup of genes analyzed demonstrated disparate expression in response to APE1 reduction in additional patient‐derived PDAC cells. Four selected genes exhibited dose‐dependent decrease in expression in response to treatment with APE1 redox inhibitor APX3330, establishing the role of APE1 redox activity in regulating their expression.

This is the first report, to our knowledge, using single‐cell RNA‐seq in a low‐passage patient‐derived tumor cell with siRNA knockdown. Furthermore, this technology provides us a way to understand the effects of knocking down a protein, APE1 with transcriptional regulation and DNA repair activities more completely as we can assess APE1 levels in each cell and then correlate that with gene expression. Additionally, the subsequent analyses determined unique pathways altered when APE1 is knocked down, but not necessarily depleted from the cells. Using an unbiased approach to identify new, putative partners and pathways for APE1 in PDAC cells, we have identified novel targets for further study of APE1‐based combination therapies for PDAC treatment, as well as potential for additional cancer indications.

## 2 Materials and methods

### 2.1 Cell culture

Pa03C, Pa02C, Panc10.05, and Panc198 (Pa20C) were obtained from A. Maitra at The Johns Hopkins University (Jones *et al*., 2008). All cells were maintained at 37 °C in 5% CO2 and grown in DMEM (Invitrogen, Carlsbad, CA, USA) with 10% serum (Hyclone, Logan, UT, USA). Cell line identity was confirmed by DNA fingerprint analysis (IDEXX BioResearch, Columbia, MO, USA) for species and baseline short tandem repeat analysis testing in February 2017. All cell lines were 100% human and a nine‐marker short tandem repeat analysis is on file. They were also confirmed to be mycoplasma free.

### 2.2 Transfection with APE1 and scrambled siRNA

The siRNA used were scrambled (SCR) (5′ CCAUGAGGUCAGCAUGGUCUG 3′, 5′ GACCAUGCUGACCUCAUGGAA 3′) and siAPE1 (5′ GUCUGGUACGACUGGAGUACC 3′, 5′ UACUCCAGUCGUACCAGACCU 3′). All siRNA transfections were performed as previously described (Fan *et al*., 2003; Fishel *et al*., 2008, 2010; Logsdon *et al*., 2016; Wang *et al*., 2004). Briefly, 1 × 10^5^ cells are plated per well of a six‐well plate and allowed to attach overnight. The next day, Lipofectamine RNAiMAX reagent (Invitrogen) was used to transfect in the APE1 and SCR siRNA at concentrations between 10 and 50 nm following the manufacturer's indicated protocol. Opti‐MEM, siRNA, and Lipofectamine were left on the cells for 16 h, and then, regular DMEM with 10% serum were added. Cells were assayed for RNA and protein expression 3 days following transfection.

### 2.3 APX3330 treatment

APX3330 was prepared as previously described (Fishel *et al*., 2010; Su *et al*., 2011). 2 × 10^5^ Pa02C cells are plated per well of a six‐well plate and allowed to attach overnight. The next day, APX3330 in low‐serum (2%) DMEM at 20 and 40 μm was added to the wells. DMSO was used as the vehicle control. Cells were treated for 24 h, after which they were collected for RNA expression analysis.

### 2.4 Western blot analysis

For whole‐cell lysates, cells were harvested, then lysed in RIPA buffer (Santa Cruz Biotechnology, Santa Cruz, CA, USA), and protein was quantified and electrophoresed. Immunoblotting was performed using the following antibodies: APE1 (Novus Biologicals, Littleton, CO, USA) and vinculin (Sigma, St. Louis, MO, USA). For qRT‐PCR experiments, APE1 expression was at least 80% decreased compared to scrambled control in order to be considered for further analysis.

### 2.5 Single‐cell RNA sequencing

Three days post‐transfection, SCR/siAPE1 cells were collected and loaded into 96‐well microfluidic C1 Fluidigm array (Fluidigm, South San Francisco, CA, USA). All chambers were visually assessed and any chamber containing dead or multiple cells was excluded. The SMARTer system (Clontech, Mountain View, CA, USA) was used to generate cDNA from captured single cells. The double‐stranded complimentary DNA quantity and quality were assessed using an Agilent Bioanalyzer (Agilent Technologies, Santa Clara, CA, USA) with the High Sensitivity DNA Chip. A total of 48 SCR and 48 siAPE1 cells were chosen for sequencing. The Purdue Genomics Facility prepared libraries using a Nextera kit (Illumina, San Diego, CA, USA). Unstranded 2 × 100 bp reads were sequenced using the HiSeq2500 (Illumina, San Diego, CA, USA) on rapid run mode in one lane. RNA‐seq data are available at the Gene Expression Omnibus (GEO) through accession number GSE99305.

### 2.6 Bioinformatics and statistical analyses

Read quality was observed using fastqc v. 0.11.2 (Andrews, 2010), and then, quality trimming was performed using fastx‐Toolkit v. 0.0.13.2 (Gordon, 2009). A fastx trimscore of 30 and a trim length of 50 were used. tophat2 (Kim *et al*., 2013; Trapnell *et al*., 2009) was used to align trimmed reads to the human genome (ENSEMBL version GrCh38.p7). One mismatch was allowed. The htseq‐count script in htseq v.0.6.1 (Anders *et al*., 2015) was run to count the number of reads mapping to each gene. htseq used biopython v.2.7.3 in the analysis. In order to determine which genes were differentially expressed, we used the r package BPSC (Vu *et al*., 2016), which is specifically designed to analyze single‐cell RNA‐seq data.

In order to facilitate comparison of the additional experimental designs in this study, the explicit mathematical expression of the linear component of our generalized model used for the baseline differential expression analysis is given by the following:

$$\mu_{ij}=\beta_{0j}+\beta_{1j}I{\left[\text{siAPE1}\right]}_{i}$$

where μ_*ij*_ is the expected value of the beta‐Poisson count distribution of the *i*th cell for the *j*th gene, β_0_ is the intercept, and β_1_ is the gene expression in log(counts per million). The expression *I*[siAPE1]_*i*_ is an indicator variable that takes the value of one when a cell belongs to the siAPE1‐knockdown group. We can then test for differential expression of the *j*th gene using the null (denoted as *H*_0_) and alternative (denoted as *H*_1_) hypotheses as follows:

$$H_{0}:\beta_{1j}=0$$

$$H_{1}:\beta_{1j}\neq 0$$

Furthermore, scRNA‐seq allowed us to separate the siAPE1 cells as having either undetectable APE1 (defined as a cell with zero expression of APE1) or detectable APE1 (defined as a cell with greater than zero expression of APE1). The model for analyzing these three categories is given by

$$\begin{array}{cc} \mu_{ij} & =\beta_{0j}+\beta_{1j}I{\left[\text{siAPE1}\right]}_{i}I{\left[\text{APE1}>0\right]}_{i}\\ & \;+\beta_{2j}I{\left[\text{siAPE1}\right]}_{i}I{\left[\text{APE1}=0\right]}_{i} \end{array}$$

where the expression *I*[siAPE1]_*i*_ *I*[APE1 > 0]_*i*_ takes the value of one when the *i*th cell both belongs to the siAPE1 group and has nonzero APE1 expression (detectable siAPE1). The expression *I*[siAPE1]_*i*_ *I*[APE1 > 0]_*i*_ takes the value of one when the *i*th cell belongs to the siAPE1 group and has no detectable expression of APE1 (undetectable siAPE1). A test for differential expression of the *j*th gene was performed using the null and alternative hypotheses

$$H_{0}:\beta_{1j}=0,\;\beta_{2j}=0$$

$$H_{1}:\text{Atleast}\;\text{one}\;\text{of}\;\beta_{1j}\neq 0\;\text{or}\;\beta_{2j}\neq 0$$

This model has two parameters that can be tested for joint significance, whereas the initial SCR/siAPE1 model only had one parameter to test. While it is possible to estimate the joint significance with a single test of both parameters, we computed the parameter‐specific significance in order to gain insight into the individual differences between undetectable siAPE1 and detectable siAPE1 groups with respect to the SCR control. In practice, we tested each of these parameters separately and reported their joint significance of the resulting *P*‐values using Fisher's method (Fisher, 1925). For two *P*‐values *P*_1*j*_, *P*_2*j*_ corresponding to test of β_1*j*_, β_2*j*_ for the *j*th gene, the combined test statistic is described as

$$F=-2\times \text{log}\left(P_{1j}\right)-2\times \text{log}\left(P_{2j}\right)∼\chi_{4}^{2}$$

where *F* is distributed as a chi‐squared random variable with four degrees of freedom under the null hypothesis. The combined *P*‐value P* is therefore computed as

$$P^{*}=1-P\chi_{4}^{2}\left(F^{*}<F\right)$$

where $P\chi_{4}^{2}$ denotes the cumulative distribution function of a $\chi_{4}^{2}$ random variable and F* is the empirical test statistic computed similar to *F* above, only using the computed *P*‐values for each gene.

Ingenuity pathway analysis (IPA) was utilized in performing network analyses (QIAGEN Redwood City, www.qiagen.com/ingenuity). An upstream regulator analysis, canonical pathway analysis, mechanistic networks analysis, causal network analysis, and downstream effects analysis were performed using IPA (results were deemed significant for *P* < 0.05). Algorithms and details of each type of network analysis are presented in Kramer *et al*. (2014).

### 2.7 Quantitative real‐time PCR

Quantitative real‐time PCR was used to measure the mRNA expression levels of the various genes identified from the scRNA‐seq analysis. Following transfection, total RNA was extracted from cells using the Qiagen RNeasy Mini kit (Qiagen, Valencia, CA, USA) according to the manufacturer's instructions. First‐strand cDNA was obtained from RNA using random hexamers and MultiScribe reverse transcriptase (Applied Biosystems, Foster City, CA, USA). Quantitative PCR was performed using SYBR Green Real Time PCR master mix (Applied Biosystems) in a CFX96 Real Time detection system (Bio‐Rad, Hercules, CA, USA). The relative quantitative mRNA level was determined using the comparative *C*_t_ method using ribosomal protein L6 (RPL6) (Pa03C) or actin (Panc10.05, Panc 198, Pa02C) as the reference gene. The primers used for qRT‐PCR are detailed in Table S1. Experiments were performed in at least triplicate for each sample. Statistical analysis performed using the $2^{-\Delta \Delta C_{T}}$ method and analysis of covariance (ANCOVA) models, as previously published (Fishel *et al*., 2015).

## 3 Results

### 3.1 scRNA‐seq Analysis of APE1‐knockdown cells

**Figure 1:**
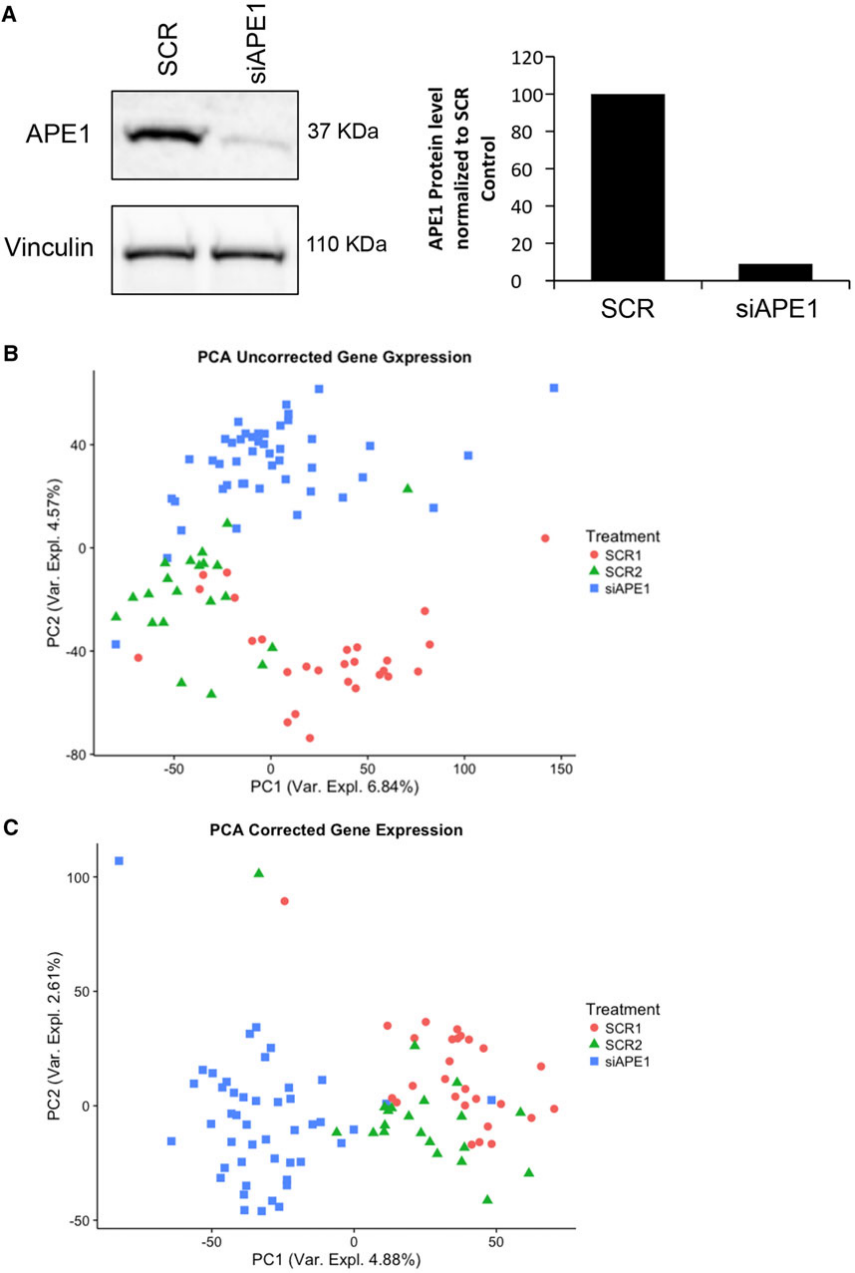
APE1 expression and batch effects in cells following siRNA knockdown. (A) Representative western blot and densitometry analysis of Pa03C cells following APE1 knockdown using 20 nm siRNA. Vinculin is used as loading control. siAPE1 samples had 10% APE1 levels in comparison with the SCR control sample. (B) Principal components analysis of uncorrected gene expression data. (C) Principal components analysis of corrected gene expression data. Following corrections for batch effects using cell cycle‐annotated genes, the SCR1 and SCR2 groups come together along the *x*‐axis to form a single SCR group.

The siRNA knockdown of APE1 does not result in complete loss of the APE1 protein, as detected by western blotting, with 10–20% APE1 protein expression observed in the siAPE1 samples compared to the scrambled controls (SCR) as shown by representative western blot shown in Fig. 1A. We used 20 nm siRNA as levels greater than this result in off‐target effects and cell killing not related to APE1 functions. Therefore, in order to clearly identify changes in gene expression specifically related to the amount of APE1 protein within each individual cell, single‐cell RNA‐seq was performed on cells following APE1 siRNA knockdown.

### 3.2 Correcting for batch effects using cell cycle‐annotated genes

Due to sample preparation constraints, the siAPE1 and SCR cells were split across three batches, with one batch containing siAPE1 and two batches containing SCR cells (SCR1 and SCR2). Differences between cell batches were corrected by applying the scLVM r package (Buettner *et al*., 2015). In conjunction with scLVM, the Biomart r package (Durinck *et al*., 2009) was used to obtain a list of cell cycle‐annotated genes. Specifically, the Gene Ontology (GO) term GO:0007049 (Gene Ontology Consortium, 2015) was used to identify 189 genes with the annotation name of ‘cell cycle’. Of these 189 genes, only 102 coincided with the genes remaining in our analysis due to the removal of genes exhibiting low expression across all cells (gene detection rate quality control filtering). We then fit a latent variable model to account for cell cycle confounding while also incorporating treatment and control covariates into the model. Using the fitted latent variable model, it is then possible to regress out the cell cycle confounding and compute a corrected dataset. A more detailed explanation can be found in the supplementary material of Buettner *et al*. (2015).

As an illustration, the plot in Fig. 1B demonstrates the two principal components before correcting for cell cycle and shows that the most influential source of variation (i.e., the *x*‐axis representing 6.84% of the total variation) in the data corresponds to the axis along which SCR1 and SCR2 cells are separated. In contrast, the second most influential source of variation (i.e., the *y*‐axis representing 4.57% of the total variation) corresponds to the axis along which siAPE1 and SCR cells are separated.

In the principal components plot following cell cycle correction (Fig. 1C), the SCR1 and SCR2 cells show greater similarity, which results in the largest source of variation (i.e., the horizontal axis representing 4.88% of the total variation) now corresponding to the axis along which the siAPE1 and SCR cells are separated. Using cell cycle correction, we have effectively removed the variation attributed to cell cycle‐annotated genes without removing the variation attributed to the differences between siAPE1 treatment and scrambled control. Thus, the largest source of variation between the cells is now attributed to APE1 knockdown.

### 3.3 Differential expression of genes in the siAPE1‐knockdown and SCR control cells

Initially, 48 SCR cells and 48 siAPE1 cells were captured for sequencing. Two SCR and siAPE1 cells each were discarded prior to sequencing due to the presence of multiple cells in the capture site. Cell detection rates (percentage of genes detected in each cell) and gene detection rates (percentage of cells with a given gene expressed) were used for statistical quality control. A threshold of 5% was used for both detection rates, resulting in a dataset of 94 cells and 15 351 genes. With the median number reads per cell of 0.95 million, we normalized the total number of reads per cell to one million. After the aforementioned cell cycle correction was performed, a further three outlier cells were removed as they demonstrated signs of PCR bias with extremely high expression counts for some genes. After all quality control measures and the removal of outliers, the number of genes detected per cell averaged 7095.7 using the original (i.e., prior to correcting for cell cycle confounding) gene expression counts. For each gene, the average number of cells with nonzero gene counts was 42.1 using the original gene expression counts.

**Figure 2:**
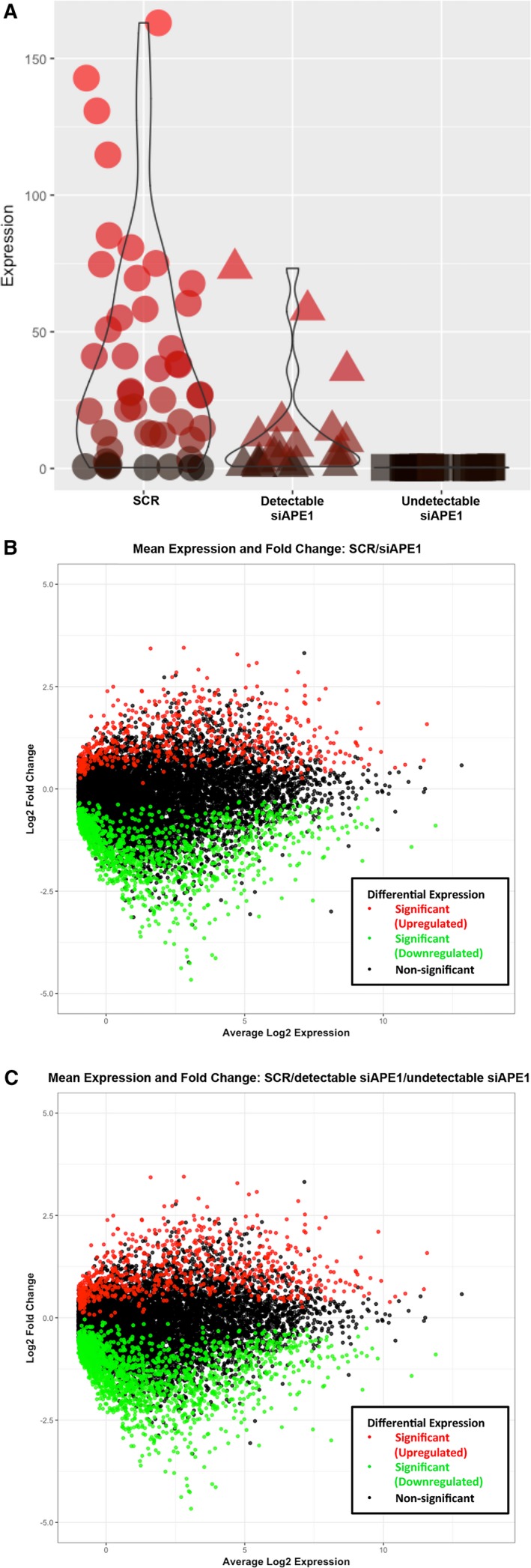
Results of scRNA‐seq and comparison of analyses. (A) Violin plot illustrating the differences in APE1 RNA expression counts per million (CPM) reads in the SCR, detectable siAPE1, and undetectable siAPE1 samples. The color bar indicates expression levels of APE1 in log2 CPM. (B) Mean expression and fold change plot using SCR and siAPE1 cells as the two groups in the analysis. Significantly DEGs in red exhibited upregulated expression, and green genes had significantly downregulated expression. (C) Mean expression and fold change plot using SCR, detectable siAPE1, and undetectable siAPE1 cells in the analysis. Note that while the analysis uses three separate groups, this plot uses SCR and siAPE1 for calculation of the mean expression and fold change due to the limitations of the graph.

The average APE1 expression in the remaining 46 cells in the SCR group was 101.6 reads per million. Of the siAPE1 cells (*n* = 45), 25 cells had no detectable APE1 expression with zero APE1 counts. The remaining 20 cells showed diminished APE1 expression, with an average of 37.7 reads per million. A violin plot showing the distribution of the cells in each of these groups can be found in Fig. 2A.

While there are many available software packages that are commonly used for differential expression analysis, there are important differences between them in terms of what assumptions are made about the distribution of the count data arising from RNA‐seq experiments. Two such r packages that use a generalized linear model in order to model non‐normally distributed data are edgeR (Robinson *et al*., 2010) and BPSC (Vu *et al*., 2016). The package edgeR models the counts with an overdispersed (larger variance) Poisson distribution (also known as the negative‐binomial distribution), which is perhaps not appropriate for single‐cell RNA‐seq data due to the fact that there are many more zero counts in these data (a phenomenon referred to as zero inflation) compared to bulk RNA‐seq. Our experimental results using edgeR resulted in a large number of DEGs, with a potentially high false discovery rate (data not shown). Alternatively, the r package BPSC models the counts in a more flexible beta‐Poisson distribution that can appropriately account for the zero inflation in the single‐cell data with the use of additional parameters in the model. Although there are other packages that are specifically developed for single‐cell differential expression, the BPSC package is relatively fast and performs well when compared to these other packages (Vu *et al*., 2016). Therefore, the BPSC r package was used in this study for the differential expression analysis between SCR cells (*n* = 46) and siAPE1‐knockdown cells (*n* = 45).

However, it is worth emphasizing that our model is more sophisticated than simple linear regression, as the distributional assumptions made about the data are fundamentally different. The equations used to generate the baseline differential expression analysis are found in Materials and methods. With this statistical design, the BPSC r package reported 1950 DEGs between the siAPE1 and SCR cells using a false discovery rate cutoff of 5%.

Of these DEGs, 71.7% had lower expression levels in the siAPE1 cells. In comparison, 58.5% of all genes sequenced had lower expression in the siAPE1 cells, although many of these genes had very low expression overall (Fig. 2B). Using Fisher's exact test (Fisher, 1922) on the number of genes with statistically significantly increased/decreased expression vs genes with nonsignificant changes in expression, we obtain a *P*‐value of 10^−16^, a highly significant result. This indicates that the predominantly inhibitory effect of APE1 knockdown on the DEGs is greater than any global decrease in expression that may be caused due to external factors (such as cell viability).

### 3.4 Identifying differentially expressed genes in relation to APE1 levels within the cell

One of the advantages of performing scRNA‐seq is that it allows us to look at APE1 expression in each individual cell. It is therefore possible to use this information to categorize cells within the siAPE1 group as having either undetectable APE1 (defined as a cell with zero expression of APE1) or detectable APE1 (defined as a cell with greater than zero expression of APE1). As previously mentioned, within the siAPE1 group, there were 25 cells with undetectable APE1 (hereafter called undetectable siAPE1) and 20 cells exhibiting detectable but reduced APE1 expression (hereafter called detectable siAPE1).

The delineation of the siAPE1 cells allowed us to consider the SCR control, detectable siAPE1, and undetectable siAPE1 cells as three different categories. Such a model is appropriate if we consider detectable siAPE1 cells to be distinct from undetectable siAPE1 as well as SCR control cells.

This analysis allows us to detect differences that may be present between SCR and detectable siAPE1 cells, between SCR and undetectable siAPE1 cells, or between SCR and both categories of siAPE1 cells. We found that this joint analysis guards against a single outlier preventing a gene from being reported as differentially expressed as one parameter may be reported as insignificant but not the other. Additionally, because the direction of the expression change in the DEGs is expected to be consistent as we move from the SCR group to the detectable siAPE1 group to undetectable siAPE1 group, this experimental design aids in the interpretation of results and helps to identify genes potentially affected by outliers. This SCR/detectable siAPE1/undetectable siAPE1 analysis identified 2837 genes using a false discovery rate of 5%. Of the 1950 DEGs identified in the SCR/siAPE1 analysis, 1945 (99.7%) were found to be differentially expressed in this subsequent analysis. Additionally, 72.1% of the DEGs were downregulated (Fig. 2C), similar to the 71.7% downregulated in the SCR/siAPE1 analysis. This consistency indicated that the increase in number of DEGs identified was due to the more rigorous statistical model, making it the preferable analysis.

**Figure 3:**
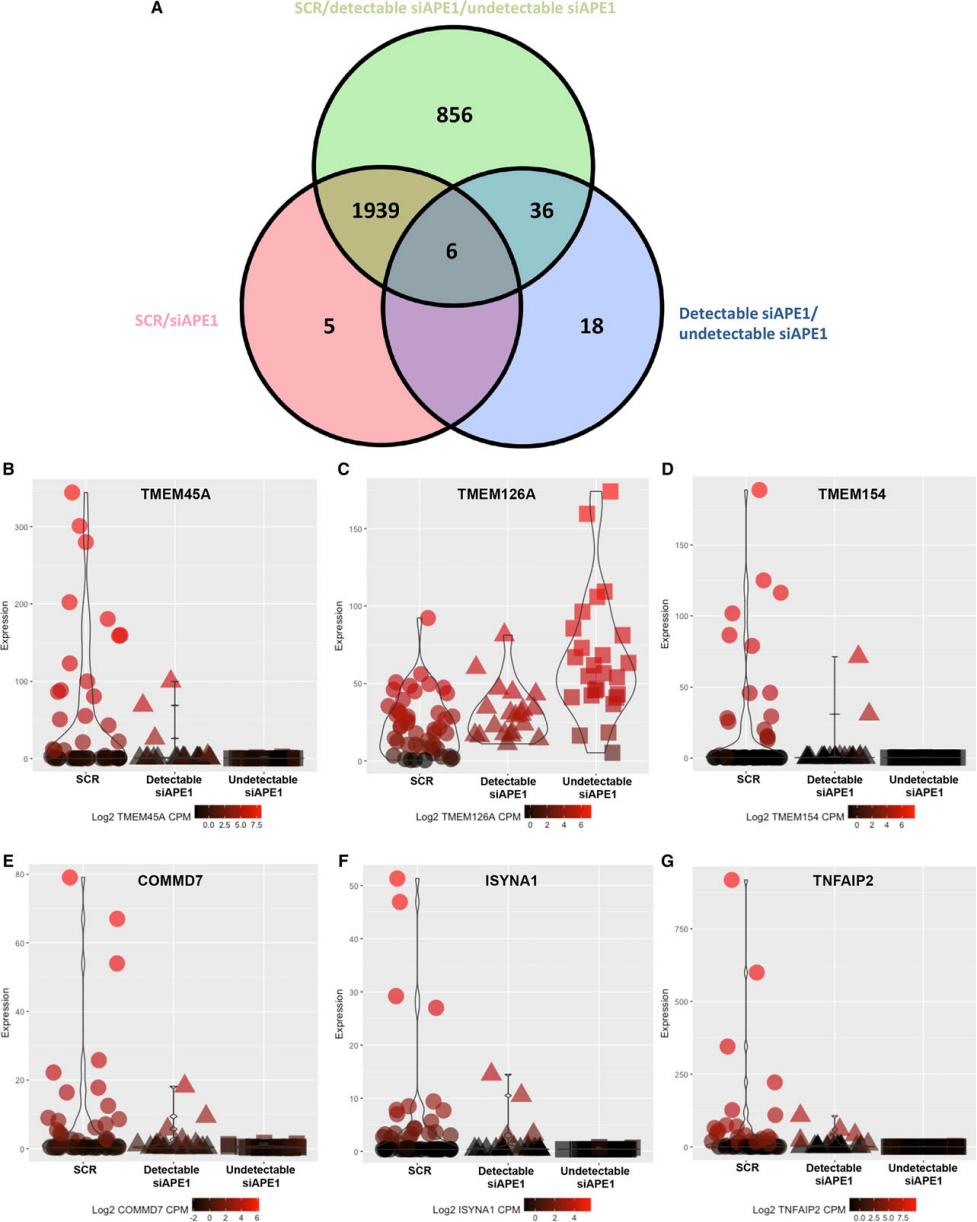
Identifying DEGs in relation to APE1 levels. (A) Venn diagram showing the three analyses performed on the scRNA‐seq data and the overlapping genes between them. Six genes were significantly changed in all three analyses, (B) TMEM45A, (C) TMEM126A, (D) TMEM154, (E) COMMD7, (F) ISYNA1, and (G) TNFAIP2. These genes show increased changes in expression as APE1 levels are reduced further from SCR to detectable (but reduced) siAPE1 to undetectable siAPE1.

Analysis was also performed to investigate which genes were differentially expressed between the detectable and undetectable siAPE1 cells. This analysis is statistically underpowered due to the smaller sample size of the two cell groups. It resulted in only 60 DEGs being identified, indicating that the detectable and undetectable siAPE1 cells had similar gene expression patterns, especially when compared to the SCR control cells. When comparing the DEGs to the SCR/detectable siAPE1/undetectable siAPE1 results, 42 genes were found to overlap, while only six genes overlapped with the SCR/siAPE1 analysis (Fig. 3A). These six genes (*TMEM45A*,* TMEM126A*,* TMEM154*,* COMMD7*,* ISYNA1*, and *TNFAIP2*) were the only genes overlapping between all three analyses. Violin plots illustrating the expression of these genes in relation to APE1 expression per cell are shown in Fig. 3B–G. The presence of these six genes in all three analyses confirms that as APE1 levels decrease, the expression levels of these six genes change further.

### 3.5 Determining the clinical relevance of the differentially expressed genes

One overarching objective of our studies is to ascertain potential combinations of APE1 inhibition with clinically approved drugs that impinge on pathways impacted by altered APE1 expression, initially in pancreatic cancer, but eventually in other cancers. Toward this goal, the clinical relevance of the DEGs identified by the different analyses was investigated using TCGA (Cancer Genome Atlas Research Network *et al*., 2013), which contains data such as tumor gene expression and clinical outcomes from patients with cancer. Due to the small number of DEGs identified in the detectable siAPE1/undetectable siAPE1 analysis, it was excluded from this TCGA analysis. Both the SCR/siAPE1 and SCR/detectable siAPE1/undetectable siAPE1 analyses were utilized. Performing this TCGA analysis allowed us to measure the clinical relevance of the DEGs identified in this study, and also provided a performance metric for the two analyses.

We used the RTCGA toolbox (Samur, 2014) to analyze the data from the TCGA. In this analysis, a gene is defined as clinically relevant if its expression level at the time of sequencing is statistically significantly related to the number of days until death in patients with pancreatic cancer. The statistical significance of a gene is determined using the Cox proportional hazards regression model (Cox, 1972), a commonly used model in clinical trials and biostatistics. Specifically, we regressed the outcome of days until death (accounting for censoring due to a patient still being alive at the time of sequencing) on the normalized gene expression data of patient tumor samples via bulk RNA‐seq using the r package survival (Therneau, 2015). We chose to include only expression levels in our analysis, modeling one gene at a time across all tumor types and stages. In all, we included 178 patient tumor samples and considered a total of 20 501 genes. Due to naming conventions and quality control procedures, only 10 292 were in common between the total number of genes sequenced in our scRNA‐seq analysis and the TCGA analysis of survival outcomes. Therefore, for this analysis, we limit our discussion to only these 10 292 genes. For this reason, the total number of DEGs reported below for both our differential expression analyses are fewer than reported in previous sections.

The TCGA analysis resulted in 1627 genes statistically significantly related to time until death using a false discovery rate of 5%. Of the 1486 DEGs considered from our SCR/siAPE1 analysis, 246 genes (16.6%) were found to be clinically relevant. The SCR/detectable siAPE1/undetectable siAPE1 analysis identified 345 clinically relevant genes (16.3%) of the available 2115 DEGs. The full results are available online in the Gene Expression Onmibus (GEO), accession number GSE99305.

The SCR/detectable siAPE1/undetectable siAPE1 analysis identified more DEGs that are clinically relevant without a change in the overall percentage of clinically relevant genes. This further illustrates that the 856 genes unique to the analysis are not statistical anomalies but authentic results identified due to a more stringent statistical model. Because of this result, all following analyses were carried out using the SCR/detectable siAPE1/undetectable siAPE1 results.

### 3.6 Gene expression patterns in cancer‐related pathways

**Figure 4:**
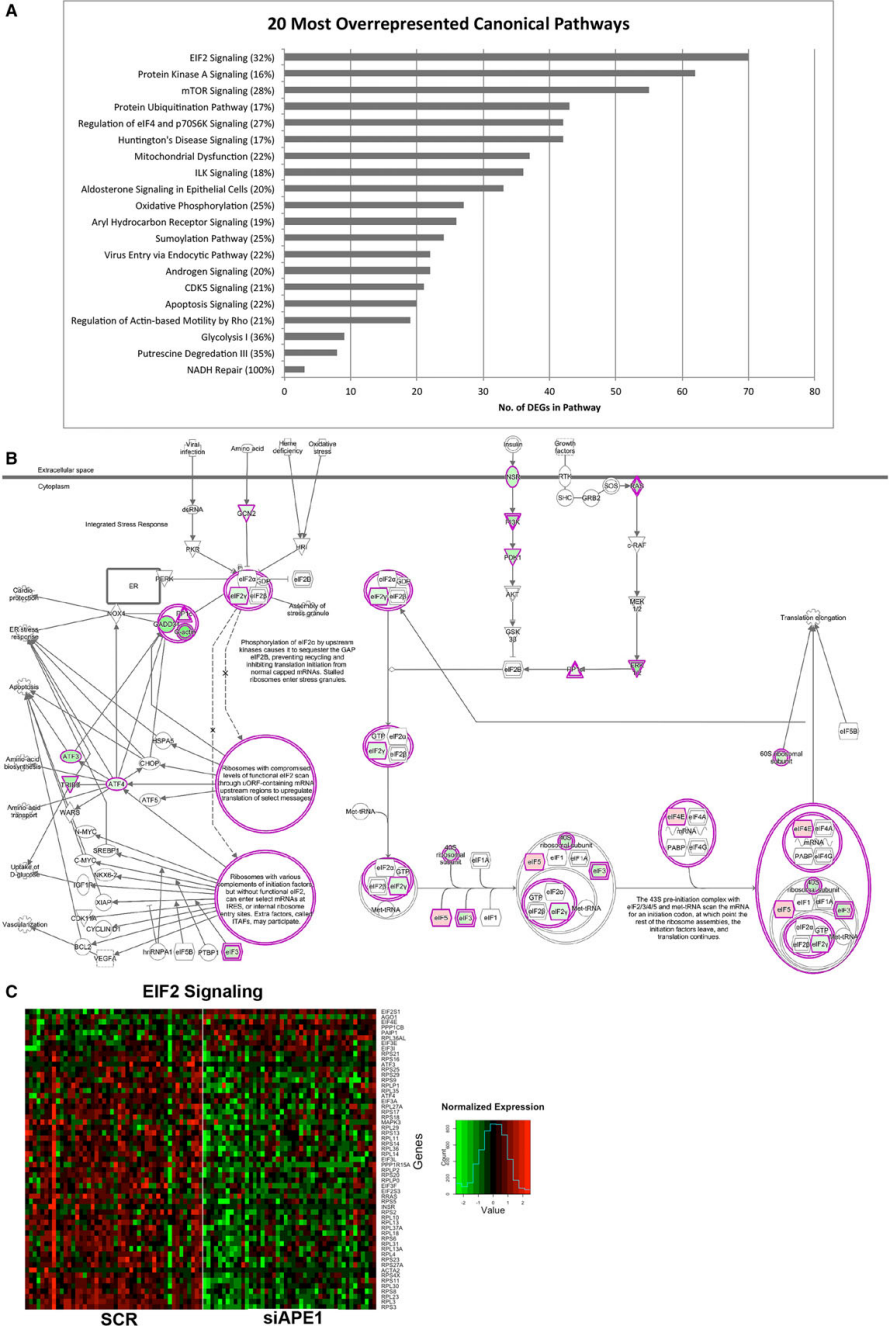
Overrepresented canonical pathways. (A) The 20 most significantly overrepresented pathways following IPA on the SCR/detectable siAPE1/undetectable siAPE1 results are shown. The *x*‐axis shows the number of genes that were differentially expressed in the overrepresented pathways. The percentages next to the pathway labels on the *y*‐axis show the percentage of genes in the pathway which are differentially expressed between SCR and siAPE1 cells. (B) Changes in the EIF2 pathway. The EIF2 pathway was the pathway most affected by APE1 knockdown with 70 DEGs. Genes that are upregulated in siAPE1 cells are shown in pink, whereas those that are downregulated are shown in green. Genes or complexes that were identified as differentially expressed are outlined in pink. (C) Heatmap showing changes in expression of DEGs per cell involved in the EIF2 pathway. Box showing colors corresponding to normalized changes in expression shown, with green indicating downregulation and red signifying upregulated genes.

Ingenuity pathway analysis was used to determine pathways regulated by APE1 based on the DEGs previously identified in the SCR/detectable siAPE1/undetectable siAPE1 analysis. Full pathway analysis results are in Table S2. A total of 104 canonical pathways were identified as overrepresented using a one‐tailed Fisher's exact test (Fisher, 1922). Data presented in Fig. 4A demonstrate the 20 most statistically significant overrepresented pathways, six of which were previously unlinked to APE1. The EIF2 signaling pathway (*P* = 1.58 × 10^−18^) with 70 DEGs was found to be the pathway most affected by APE1 knockdown. An overview of the pathway with the genes that were affected is presented in Fig. 4B, with a heatmap highlighting the 70 DEGs, and their expression in each cell is shown in Fig. 4C. Other previously unlinked pathways were the mTor pathway (*P* = 3.98 × 10^−12^) with 55 DEGs and the regulation of eIF4 and p7056K signaling pathways (*P* = 3.63 × 10^−9^) with 42 DEGs. These pathways, along with the virus entry via endocytic pathway, regulation of actin‐based motility by Rho and putrescine degradation pathways, are now putatively linked to APE1 based on our scRNA‐seq data, expanding APE1's already diverse role within the cell. In total, 44 pathways previously unassociated with APE1 were identified in this study. These results highlight the importance of single‐cell RNA‐seq in determining clear gene expression and pathway interactions.

A number of the significant pathways affected by APE1 knockdown confirm previous observations and therefore provided validation for the results. For example, the HIF1α signaling pathway, shown to be regulated by APE1 (Lando *et al*., 2000), was found to be significantly downregulated in the pathway analysis (*P* = 0.006). Similarly, the mitochondrial dysfunction (*P* = 8.12 × 10^−6^) and Huntington's disease signaling (*P* = 4.07 × 10^−4^) pathways are both in the top ten significantly overrepresented pathways affected by APE1 knockdown. The mitochondrial dysfunction pathway has 37 DEGs, while there are 42 DEGs in the Huntington's disease signaling pathway. Mitochondrial dysfunction is believed to play a role in Huntington's disease pathology, and prior studies have demonstrated that APE1 is important for the maintenance of mitochondrial function (Li *et al*., 2012; Siddiqui *et al*., 2012). APE1 is also known to participate in mitochondrial DNA repair functions (Ballista‐Hernandez *et al*., 2017; Stuart *et al*., 2004; Vascotto *et al*., 2009). While APE1 is known to influence these pathways, this study expands our understanding of APE1 within the cell by implicating the genes in the pathways that are affected by APE1 knockdown.

### 3.7 Validating scRNA‐seq results using qRT‐PCR

**Figure 5:**
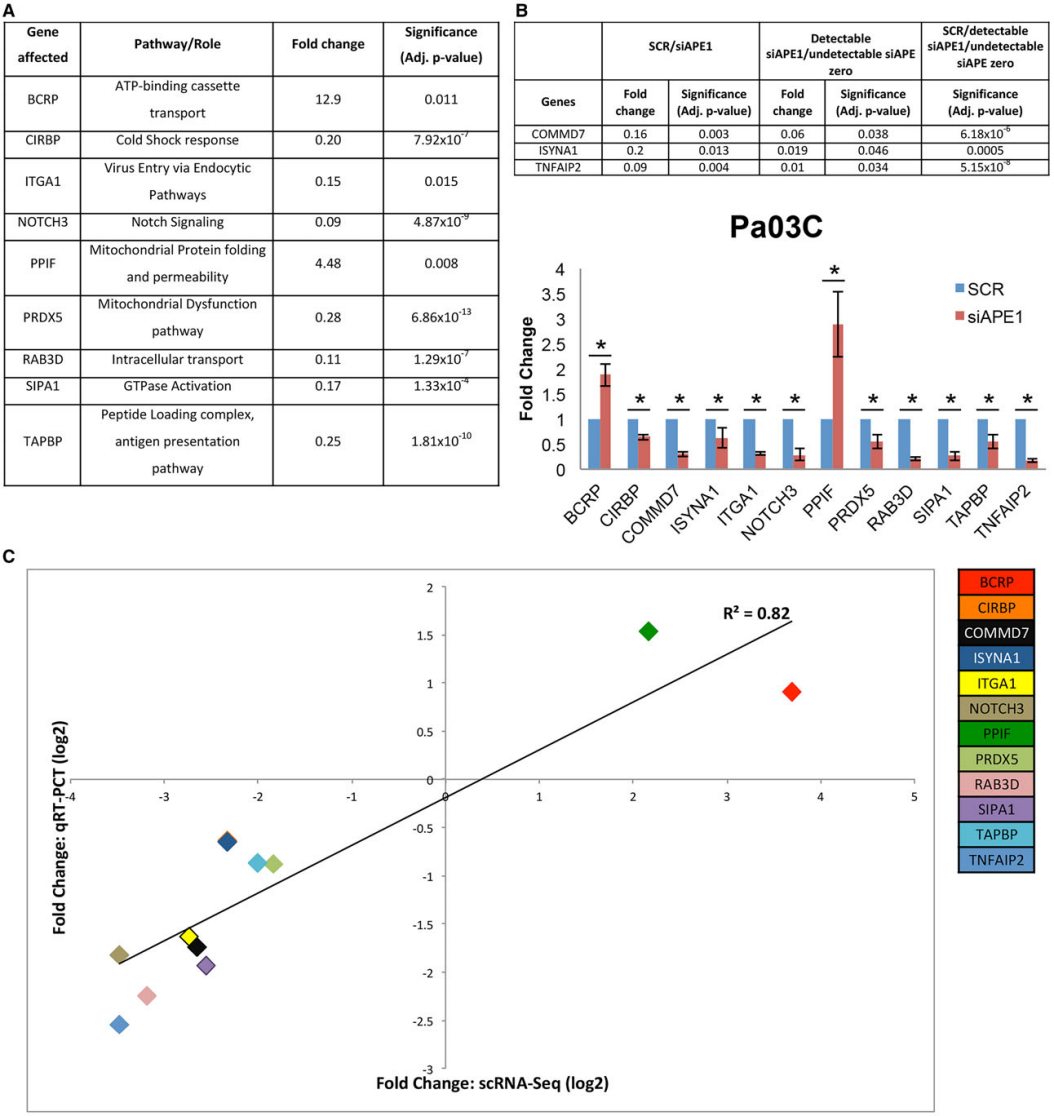
Validation of scRNA‐Seq by qRT‐PCR in Pa03C cells. (A) Genes chosen for qRT‐PCR validation following SCR/siAPE1 validation. (B) Genes statistically significant in all three analyses chosen for qRT‐PCR validation. (C) Expression of selected genes assessed via qRT‐PCR in Pa03C cells. The cells were collected after siRNA knockdown and assessed for a reduction in APE1 protein levels of 80% or greater. Each graph is the result of three independent experiments, showing average fold change in siAPE samples compared to SCR ± SD. *P < 0.05 (ANCOVA model). (D) Validation analysis. Relation between log2 fold changes following scRNA‐Seq (*x*‐axis) and qRT‐PCR (*y*‐axis). *R* ^2^ = 0.82. Linear regression analysis of the slope provided *P* < 0.0001. The 12 genes used for validation are color‐coded.

The scRNA‐seq results of the SCR/siAPE1 analysis were validated by performing qRT‐PCR in Pa03C cells following siRNA knockdown. A panel of genes, from distinct pathways and showing varying changes following knockdown, was chosen (Fig. 5A). These genes were present in both the SCR/siAPE1 and SCR/detectable siAPE1/undetectable siAPE1 analyses. Efficiency of siRNA knockdown was assessed using western blots, with only samples exhibiting greater than 80% reduction in APE1 expression compared to the scrambled controls chosen.

In addition, validation of three genes that were differentially expressed and statistically significant in all analyses (SCR/siAPE1, detectable siAPE1/undetectable siAPE1, and SCR/detectable siAPE1/undetectable siAPE1) was performed. The presence of these genes (Fig. 5B) within all three analyses indicates that their expression changes more dramatically with greater APE1 knockdown.

The genes that showed statistically significantly increased or decreased expression in scRNA‐seq exhibit changes in the same direction following qRT‐PCR (Fig. 5C), with a decrease seen in the mRNA levels of *CIRBP*,* COMMD7*,* ISYNA1*,* ITGA1*,* NOTCH3*,* PRDX5*,* RAB3D*,* SIPA1*,* TAPBP*, and *TNFAIP2*. The expression of breast cancer resistance protein (*BCRP*) and *PPIF* was significantly increased following knockdown. We plotted the fold changes from scRNA‐seq against qRT‐PCR fold changes in Fig. 5D. With an *R* ^2^ value of 0.82 and *P* < 0.0001 (linear regression analysis), we confirmed that the fold changes were consistent and validated the single‐cell scRNA‐seq studies.

### 3.8 Differences in gene expression of PDAC cell lines in response to APE1 siRNA knockdown

**Figure 6:**
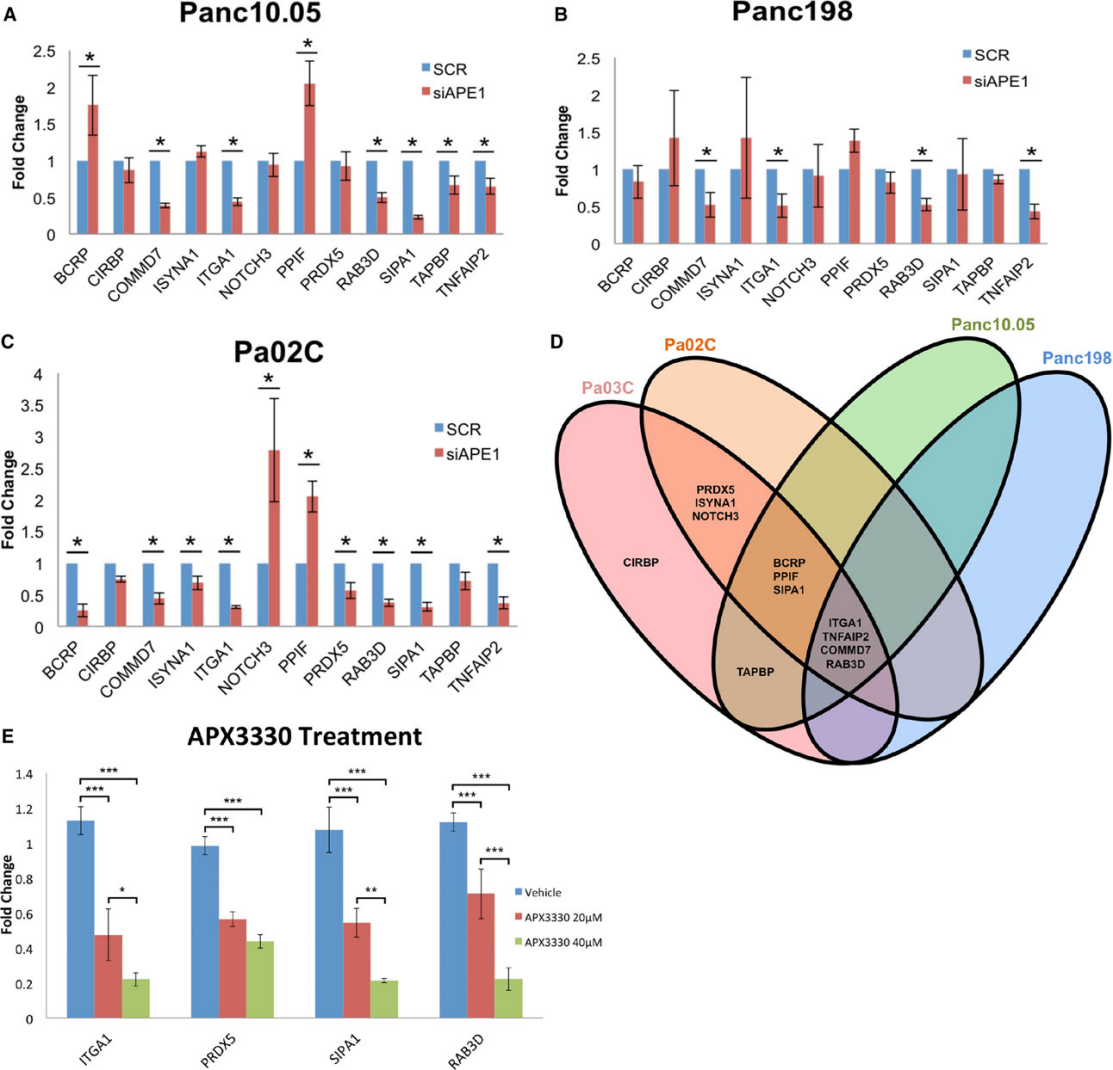
Different PDAC cell lines exhibit varied changes to expression of select genes following siRNA knockdown and APX3330 treatment. Expression of selected genes assessed via qRT‐PCR in (A) Panc10.05 cells (B) Panc198 cells and (C) Pa02C cells. The cells were collected after siRNA knockdown and assessed for a reduction in APE1 protein levels of 80% or greater. Each graph is the result of at least three independent experiments, showing average fold change in siAPE samples compared to SCR ± SD. *P < 0.05 (ANCOVA model). (D) Venn diagram showing the overlapping results of qRT‐PCR between the four different PDAC cell lines. (E) Expression of genes assessed via qRT‐PCR following 24‐hr APX3330 treatment in Pa02C cells. The graph is a result of three independent experiments, showing average fold change in vehicle‐ and APX3330‐treated samples compared to naïve control ± SD. *P < 0.05, **P < 0.005, ***P < 0.001 (two‐way ANOVA applying Tukey's *post hoc* multiple comparisons test).

We proceeded to look at the effect of APE1 siRNA knockdowns in other PDAC low‐passage patient‐derived cells. The effect of APE1 knockdown on these genes varied between the different patient lines, as shown in Fig. 6.

Panc10.05 cells, derived from a primary PDAC tumor, exhibited similar results to Pa03C cells, with eight of the 12 genes showing similar changes in expression (Fig. 6A). *COMMD7*,* ITGA1*,* RAB3D*,* SIPA1*,* TAPBP*, and *TNFAIP2* show decreased expression, while *BCRP* and *PPIF* show increased expression. In contrast, *CIRBP*,* ISYNA1*,* NOTCH3*, and *PRDX5* show no change in expression in the Panc10.05 cells.

Panc198 cells, also originating from a primary tumor, produced the most varied results (Fig. 6B). *COMMD7*,* ITGA1*,* RAB3D*, and *TNFAIP2* all showed significantly decreased expression, while no change in expression was seen for *BCRP*,* CIRBP*,* ISYNA1*,* NOTCH3*,* PRDX5*,* PPIF*,* SIPA1*, and *TAPBP*.

Pa02C, a cell line generated from liver metastasis of a patient with PDAC, showed generally similar gene expression patterns to the Pa03C cells, which were also isolated from PDAC liver metastasis. In Pa02C cells, *BCRP*,* COMMD7*,* ISYNA1*,* ITGA1*,* PRDX5*,* RAB3D*,* SIPA1*, and *TNFAIP2* all demonstrated a decrease in expression, while *NOTCH3* and *PPIF* were significantly increased following knockdown (Fig. 6C). Interestingly, while changes in expression of *BCRP* and *NOTCH3* were significant, they were in opposing directions to the changes seen in Pa03C cells.

*COMMD7*,* ITGA1*,* RAB3D*, and *TNFAIP2* were significantly changed in all four cell lines (Fig. 6D). *PPIF* and *SIPA1* were differentially expressed in Pa03C, Pa02C, and Panc10.05 cells. *TAPBP* was differentially expressed in Pa03C and Panc10.05. *PRDX5*,* ISYNA1*,* BCRP*, and *NOTCH3* were common between Pa03C and Pa02C (with *BCRP* and *NOTCH3* changing in opposite directions between the cell lines), while *CIRBP* was only differentially expressed in Pa03Cs.

### 3.9 Role of APE1 redox activity in differential gene expression

The multifunctional nature of APE1 means that the differential expression observed may be either in response to altered APE1 redox signaling or BER activity. In order to isolate the impact of reduced APE1 redox signaling, Pa02C cells were treated with the specific APE1 redox signaling inhibitor APX3330. *ITGA1*,* PRDX5*,* SIPA1*, and *RAB3D* were analyzed, chosen for their importance in pathways identified in the IPA as well as pathways previously linked to APE1. All four genes tested demonstrated significant dose‐dependent decreases in gene expression when treated with APX3330 compared to vehicle control (Fig. 6E).

## 4 Discussion

In this study, we used single‐cell RNA‐seq to examine the effects of APE1 knockdown in patient‐derived PDAC cells. Generating a significant amount of data, we initially corrected for batch effects using cell cycle‐annotated genes. As detailed in Hicks *et al*. (2015), scRNA‐seq data often have batch effects that can potentially confound the results of cell type identification or, more applicable to this study, differential gene expression. Without correction, such improper statistical design in testing the difference between the two groups would lead to an increased number of false positives in any further analyses.

Initial analyses looked for DEGs comparing the SCR (*n* = 46) and siAPE1 (*n* = 45) cells regardless of APE1 expression level. This SCR/siAPE1 analysis resulted in 1950 DEGs (Fig. 2B) and allowed us to identify several new genes and pathways impacted by APE1, including the EIF2 signaling and mTOR pathways and a significant number of mitochondrial‐related genes and pathways. However, employing scRNA‐seq meant that we could further analyze the data without the limitations of bulk RNA studies. By assessing the APE1 expression in each cell, we were able to identify 25 cells in the siAPE1 group that expressed no detectable APE1 (undetectable siAPE1), while the remaining 20 cells expressed APE1 albeit at reduced levels compared to the SCR cells (detectable siAPE1). Subsequently, this afforded us the opportunity to perform a novel second analysis, in which we identified DEGs between SCR cells and cells with detectable APE1 as well as cells with undetectable siAPE1, resulting in 2837 DEGs (Fig. 2C).

A third analysis was performed comparing the detectable siAPE1 and undetectable siAPE1 cells. Only 60 genes were identified as differentially expressed between these two groups, indicating their similar gene expression patterns. Six of these genes overlapped with the previous two analyses and were the only genes in common between the three analyses (Fig. 3A). Thirty‐six DEGs overlapped between the SCR/detectable siAPE1/undetectable siAPE1 and detectable siAPE1/undetectable siAPE1 analyses. The appearance of 18 genes unique to the detectable siAPE1/undetectable siAPE1 analysis is attributed to outliers and their outsized effect on the analysis due to the smaller sample size of the analysis.

Only six genes overlapped between the three analyses (*TMEM45A*,* TMEM126A*,* TMEM154*,* COMMD7*,* ISYNA1*, and *TNFAIP2*) (Fig. 3A), demonstrating that only these six genes were further affected as APE1 levels decreased. This was an unexpected result, as we expected a larger number of genes to change further as APE1 levels decrease. Consequently, our results indicate that the change in expression of most genes following APE1 knockdown is apparent when APE1 is at least reduced by 80% (based on the number of APE1 transcripts in the siAPE1 cells), and further reduction in APE1 does not significantly increase or decrease most genes further. In the case of several downregulated genes, this was because initial APE1 knockdown (detectable siAPE1 cells) already reduced their expression to near zero, which meant further reduction in APE1 (undetectable siAPE1 cells) had no effect on them.

The Cancer Genome Atlas analysis helped us to prioritize which analysis was preferable for follow‐up studies as the analysis comparing the SCR/detectable siAPE1/undetectable siAPE1 analysis identified the highest number of DEGs. We compared the results from this analysis to two previous studies looking at the effects of APE1 knockdown on gene expression in a population of cells. The first study from Vascotto *et al*. (2009) performed microarrays on RNA from long‐established HeLa cells with conditional APE1 siRNA knockdown. The gene list from this study consisted of 858 unique gene names. Following removal of genes due to different naming conventions and quality control, 643 of those 858 genes overlapped with genes considered in our analysis. The overlap between the two studies resulted in 151 DEGs, with 85 genes matching the direction of change in expression.

A second study from Illuzzi *et al*. (2017) used a locus‐specific targeting vector to generate haploinsufficiency in the established human colon cancer cell line, HCT116. The heterozygous APE1‐knockout cells expressed approximately 50% APE1 level compared to the parental line and were collected at passage five for the microarray study. However, at the time of collection, the cells were near the end of their viability. While this may influence the DEGs that were (and were not) identified in the study, we compared the results of our analysis to the 80 DEGs from this study. Forty‐four genes overlapped with the entire set of genes (15 351) considered in our analysis, with 17 genes differentially expressed in both studies. Fourteen of those were differentially expressed in the same direction.

As demonstrated by our results presented here, gene expression patterns in response to APE1 knockdown vary between four different patient‐derived PDAC cells (Fig. 6D). Therefore, it is not a surprise to see only 17.6% overlap between our scRNA‐seq results and microarray data obtained from HeLa cells, and 17.5% overlap with HCT116 cells when considering the total number of DEGs identified in each respective study. We assume some of these differences arise from differences in cancer subtypes as well as differences in APE1 expression between experimental conditions. A key point when comparing these studies to our current study is the consideration of the tumor cells utilized: We used low‐passage primary patient‐derived tumor cells, while the other studies were performed on long‐established laboratory‐based tumor cell lines which may have lost significant characteristics of human tumors. Because scRNA‐seq is a considerably more sensitive and unbiased method of detection compared to microarrays, the disparity in gene expression is most likely amplified, making our current study more accurate of the biology occurring in the cells.

The identification of the large number of DEGs in our study allowed us to look at pathways most affected by APE1 knockdown. IPA identified 104 pathways, with 60 previously linked and 44 unlinked to APE1. One previously linked pathway of particular interest is the mitochondrial dysfunction pathway. APE1 redox and repair activity has previously been shown to be responsible for maintaining and repairing mitochondrial DNA (Ballista‐Hernandez *et al*., 2017; Li *et al*., 2012; Siddiqui *et al*., 2012; Stuart *et al*., 2004; Vascotto *et al*., 2009). The results of our studies presented here identify, for the first time, the genes responsible for these functions. Among the 37 DEGs in the pathway are *COX10* and *PRDX5*, both of which are downregulated by APE1 knockdown. COX10 has been shown to be essential for assembly and stability of the mitochondrial electron transport chain complexes I and IV (Diaz *et al*., 2006), and loss of PRDX5 sensitizes the cell to apoptosis by complex I inhibitors (De Simoni *et al*., 2008; Kropotov *et al*., 2006). This opens up the potential of combining APE1‐based therapies and existing mitochondrial complex inhibitors as a novel approach to targeting cancer cells via inhibition of the mitochondrial dysfunction pathway. Further analysis into the viability of the mitochondrial dysfunction and other affected pathways identified in this study as potential APE1‐based combination therapy targets is ongoing. Additionally, these results support the role of APE1 as a node in cancer signaling (Shah *et al*., 2017).

For validation of the scRNA‐seq, 12 novel genes were chosen that have previously not been linked to APE1. Nine of the genes are present in the SCR/siAPE1 and SCR/detectable siAPE1/undetectable siAPE1 analyses (Fig. 5A), while three were present in all three analyses (Fig. 5B). Fold changes from the SCR/siAPE1 analysis were used for comparative graph and statistical analysis (Fig. 5D). While the SCR/detectable siAPE1/undetectable siAPE1 analysis takes maximal advantage of the scRNA‐seq dataset, the SCR/siAPE1 analysis comparing all SCR cells to siAPE1 cells is an experimental design that can be replicated in a laboratory setting where siRNA knockdown results in a heterogeneous population.

The qRT‐PCR results on the genes across the four different patient‐derived cell lines highlight the differences between individual patient tumors, as well as between primary and metastatic tumors (Fig. 6D). Pa03C and Pa02C, both liver metastases of patients with PDAC (Embuscado *et al*., 2005), show similar and statistically significant gene expression changes in 10 of 12 genes. However, *BCRP* and *NOTCH3* exhibited significant changes in opposite directions (Figs 5C and 6C). In the primary PDAC cell lines Panc10.05 and Panc198 (Cui *et al*., 2012), *NOTCH3* mRNA levels are unchanged following APE1 knockdown, while *BCRP* levels are significantly increased in Panc10.05 (Fig. 6A,B). Both primary cell lines exhibited mRNA expression patterns that were more similar to each other than either metastatic line, although this is a small subset of patient lines. The differing changes in expression of *BCRP* and *NOTCH3* emphasize the importance of tumor profiling and precision oncology in therapeutic strategies for PDAC, and the need to target nodal proteins like APE1 that can affect multiple pathways.

Breast cancer resistance protein/ABCG2 is an ATP‐binding cassette (ABC) transporter that is one of the proteins responsible for multidrug resistance of cancer cells (Mo and Zhang, 2012). In PDAC, high BCRP expression corresponds to carcinogenesis, tumor progression, early recurrence, and poor survival (Lee *et al*., 2012; Yuan *et al*., 2015). Several chemotherapeutic drugs are substrates for BCRP, which results in their efflux from and reduced accumulation within the cells (Mo and Zhang, 2012). An affected drug of particular interest is 5‐fluorouracil (Yuan *et al*., 2009), which is currently part of the treatment regimen for patients with PDAC (Ellenrieder *et al*., 2016). Therefore, the discovery that APE1 knockdown affects BCRP expression is crucial when looking at future drug combinations to improve survival in PDAC. Combining APE1‐targeted agents with 5‐FU in tumors genetically similar to Pa02C should respond favorably to this combination due to reduced BCRP expression. A study in colon cancer stem cells indeed demonstrated dramatically increased cell killing when 5‐FU and an inhibitor of APE1, APX3330, were used *in vivo* (Lou *et al*., 2014).

NOTCH3, a highly conserved member of the eponymous Notch signaling pathway, has been implicated in cell survival, proliferation, differentiation, development, and homeostasis (Xiao *et al*., 2016). Increased Notch3 protein levels have been identified as a prognostic marker for patients with PDAC (Mann *et al*., 2012), and lead to increased tumor invasion, metastasis, and shortened patient survival (Zhou *et al*., 2016). Because of this, Notch3 has become a target for novel cancer therapies. γ‐secretase inhibitors and DLL4‐inhibiting antibodies both target proteins upstream of Notch3, leading to the inhibition of the Notch signaling pathway (Xiao *et al*., 2016). The identification of Notch3 as being affected by APE1 opens up the possibility of combining APE1‐targeted therapies with these inhibitors to enhance (in Pa03C) or counteract (in Pa02C) the effects of APE1 inhibition on NOTCH3 expression and function in PDAC.

Of the 10 other genes validated, four of them, *COMMD7*,* ITGA1*,* RAB3D*, and *TNFAIP2*, showed decreased expression in all four patient cell lines (Fig. 6D). COMMD7 (You *et al*., 2017), ITGA1 (Boudjadi *et al*., 2013; Gulubova, 2004; Schadendorf *et al*., 1996), RAB3D (Luo *et al*., 2016; Yang *et al*., 2003), and TNFAIP2 (Chen *et al*., 2011; Jia *et al*., 2016) have all been shown to be upregulated in various cancers including PDAC. While we cannot assume these changes will be universal in all PDAC samples, this consistency suggests that some of these genes could make promising targets or biomarkers for APE1‐based therapy or combination therapies that potentially will be useful across multiple PDAC tumor subtypes and in other tumor types.

In order to begin to correlate some of the key pathways that were elucidated with the IPA with the major functions of APE1, we chose four genes to interrogate following treatment with APE1 redox inhibitor APX3330. This small‐molecule inhibitor does not affect APE1's DNA repair function (Luo *et al*., 2008), thereby allowing us to determine the result of blockade of the redox activity of APE1 while not affecting DNA repair, unlike the siRNA studies. Using APE1 redox‐specific inhibitor APX3330, we tested four genes that showed reduced expression in siAPE1 cells to examine whether this downregulation was due to impaired APE1 redox activity. These genes were chosen based on their functions in highly significant pathways from IPA, as well as pathways previously associated with APE1.

ITGA1, part of the integrin signaling and virus entry via endocytic pathways as identified by IPA, is involved in cell proliferation (Macias‐Perez *et al*., 2008) and invasion (Yang *et al*., 2015), as well as inflammation (Becker *et al*., 2013) and fibrosis (Ramos *et al*., 2012), which have all been previously linked to APE1 (Aamann *et al*., 2016; Shah *et al*., 2017).

PRDX5 is part of the mitochondrial dysfunction pathway as identified by IPA and plays a major in protecting the cell from oxidative stress (De Simoni *et al*., 2013). SIPA1 (Takahara *et al*., 2017; Zhang *et al*., 2017) and RAB3D (Yang *et al*., 2003; Zhang *et al*., 2015) are both involved in proliferation, invasion, and metastasis in multiple cancers, exhibiting similar functions to APE1. All four genes showing significantly reduced expression in a dose‐dependent manner (Fig. 6E), establishing these genes are regulated by APE1 redox activity.

However, these genes represent a fraction of the genes identified in this initial study affected by APE1 knockdown. The identification of pathways formerly unassociated with APE1, as well as known pathways exhibiting DEGs not previously linked with APE1, opens up novel targets for APE1‐based combination therapies. In fact, initial experiments targeting some of the identified pathways in combination with APE1 inhibition appear to be promising and are the basis for future studies.

## 5 Conclusions

This study takes an unbiased statistical approach to determine the effects of APE1 knockdown in PDAC cells. While it has been long known that APE1 regulates various essential transcription factors (Cardoso *et al*., 2012; Fishel *et al*., 2015; Gaiddon *et al*., 1999; Jiang *et al*., 2010; Kelley *et al*., 2012; Lando *et al*., 2000; Logsdon *et al*., 2016), the amplified effect of APE1 knockdown on downstream targets of those transcription factors has not been previously elucidated. Employing scRNA‐seq for this investigation allowed us to apply more stringent analytical models to identify 2837 DEGs in Pa03C pancreatic cancer cells. Based on this, we identified several new pathways not previously known to be modulated by APE1 levels. We also demonstrate that APE1 appears to have an effect on modifying gene expression to a threshold of around 20% APE1. Reducing APE1 levels further does not significantly impact the target genes. This demonstrates that it is not necessary to completely knockout APE1 expression in cells to accurately study APE1.

Solid tumor microenvironments, particularly PDAC, are hypoxic, which leads to stabilization of HIF1α, a transcription factor that regulates a multitude of proteins involved in cell survival, proliferation, and invasion (Masoud and Li, 2015). HIF1α transcriptional activity is also regulated by APE1 (Fishel *et al*., 2011). Similarly, STAT3 has been shown to work with HIF1α in order to activate downstream targets in various cancers (Gariboldi *et al*., 2010; Pawlus *et al*., 2014), and we have previously shown that APE1 interacts with both STAT3 and HIF1α under hypoxia (Logsdon *et al*., 2016). Single‐cell RNA‐seq under conditions of APE1 knockdown and hypoxia would aid in further dissection of the role of APE1 in regulating genes and pathways that affect PDAC survival and invasion. As a future direction, we have begun the APE1‐knockdown/hypoxia studies and anticipate the layering of those findings with these to further refine a combination pathway strategy to attack PDAC in a hypothesis‐driven approach.

One other caveat exists in the studies as presented here. We determined alteration of gene expression following APE1 knockdown. APE1, as discussed, has at least two major roles in tumor cells; redox signaling and DNA repair. Therefore, at this point in time, we acknowledge that the altered gene expression is most likely not solely due to alteration of redox signaling as some pathways may have been altered in response to reduced APE1 BER activity. While this study identifies four genes as being regulated by APE1 redox activity, this will be further addressed in future studies using the specific APE1 redox signaling inhibitor, APX3330, as well as second‐generation analogs. APX3330 is a novel, oral anticancer agent that specifically and selectively inhibits APE1 redox activity without affecting APE1 endonuclease DNA repair activity (Fishel *et al*., 2010; Luo *et al*., 2008; Su *et al*., 2011). It is the first drug to target APE1 in cancer and enters clinical trials in 2017. This brings forward the possibility of combining APX3330 with FDA‐approved drugs that target genes affected by loss of APE1 redox activity based on patient tumor profile. This will allow us to precisely target patient tumor subtypes to achieve drug‐synthetic lethality (Brunen and Bernards, 2017) in PDAC and other cancers in the future.

## Availability of data and materials

The datasets generated and analyzed during the current study are available in the GEO, accession number GSE99305.

## Author contributions

MLF designed and directed the study. MLF and FS performed the single‐cell RNA‐seq. EG and NMA analyzed the single‐cell RNA‐seq. FS and MG performed the qRT‐PCR experiments. MRK contributed to scientific discussions for designing the study and writing the manuscript. FS, EG, and NMA wrote the manuscript. All authors contributed edits and approved the final manuscript.

## Conflict of interest

MRK is the Chief Scientific Officer and Founder of Apexian Pharmaceuticals. Although not used in these studies, Apexian has licensed E3330 (APX3330) through Indiana University Research and Technology Corporation. Apexian Pharmaceuticals had neither control nor oversight of the studies or the study design, results or interpretation, or presentation of the data in this manuscript. They did not have to approve the manuscript in any way prior to its submission.

## Supporting information


**Table S1.** Primers used for qRT‐PCR.

**Table S2.** Complete results of IPA Pathway analysis.
