# Supplementary material for: APE1/Ref‐1 knockdown in pancreatic ductal adenocarcinoma – characterizing gene expression changes and identifying novel pathways using single‐cell RNA sequencing
Source: Mol Oncol. 2017 Oct 19;11(12):1711–32. doi: 10.1002/1878-0261.12138 (PMC5709621; doi:10.1002/1878-0261.12138)
Supplement: Supplementary file 1 — Table S1. Primers used for qRT‐PCR. [file MOL2-11-1711-s001.docx]

Table S1: Primers used for qRT-PCR.

| **Gene** | **Primer** | **Sequence** |
| --- | --- | --- |
| Actin | Forward | CACCATTGGCAATGAGCGGTTC |
|  | Reverse | AGGTCTTTGCGGATGTCCACGT |
| RPL6 | Forward | ATTGCTTATAGACCGGAAGCCG |
|  | Reverse | AACTTTTTCACCCGCCATCTTG |
| BCRP/ABCG2 | Forward | GTTCTCAGCAGCTCTTCGGCTT |
|  | Reverse | TCCTCCAGACACACCACGGATA |
| CIRBP | Forward | GTCAGAGTGGTGGCTACAGTG |
|  | Reverse | GCCCTCGGAGTGTGACTTAC |
| COMMD7 | Forward | GAGCAGCGAATTGGAGAAAGTGG |
|  | Reverse | TCCATCTCGTGCAGGAAGCTGT |
| ISYNA1 | Forward | GCCAGACCAAAGTCAAGTCCGT |
|  | Reverse | CTTAGAGCGGAACTGCAATGGC |
| ITGA1 | Forward | CCGAAGAGGTACTTGTTGCAGC |
|  | Reverse | GGCTTCCGTGAATGCCTCCTTT |
| NOTCH3 | Forward | CCAGATGGCTTCACCCCGC |
|  | Reverse | TCAGTTGGCATTGGCTCCAG |
| PPIF | Forward | CGACTTCACCAACCACAATGGC |
|  | Reverse | GGTGTTAGGACCAGCATTAGCC |
| PRDX5 | Forward | TGATGCCTTTGTGACTGGCGAG |
|  | Reverse | CCAAAGATGGACACCAGCGAATC |
| RAB3D | Forward | ACGTGTTGTGCCTGCTGAGGAT |
|  | Reverse | CTTCTCGCAGATGACATCCACC |
| SIPA1 | Forward | GTGTCCACGATGCTGCCTTACA |
|  | Reverse | CTTGCTGCCAGGCTCCTGGAA |
| TAPBP | Forward | GAGCCTGTTCTCATCACCATGG |
|  | Reverse | GTAGGCAAAGCTCAAGTCCAGC |
| TNFAIP2 | Forward | TGCTCCAGAACCTGCATGAGGA |
|  | Reverse | AACTCAGGCAGCCTCGTGTCTA |
