# Supplementary material for: APE1/Ref‐1 knockdown in pancreatic ductal adenocarcinoma – characterizing gene expression changes and identifying novel pathways using single‐cell RNA sequencing
Source: Mol Oncol. 2017 Oct 19;11(12):1711–32. doi: 10.1002/1878-0261.12138 (PMC5709621; doi:10.1002/1878-0261.12138)
Supplement: Supplementary file 2 — Table S2. Complete results of IPA Pathway analysis. [file MOL2-11-1711-s002.docx]

Table S2: Complete results of IPA Pathway analysis.

| **Ingenuity Canonical Pathways** | **p-value** |
| --- | --- |
| EIF2 Signaling | 1.58x10^-18^ |
| mTOR Signaling | 3.98x10^-12^ |
| Regulation of eIF4 and p70S6K Signaling | 3.63x10^-9^ |
| Mitochondrial Dysfunction | 8.12x10^-6^ |
| Oxidative Phosphorylation | 1.02x10^-5^ |
| Sumoylation Pathway | 2.63x10^-5^ |
| Aldosterone Signaling in Epithelial Cells | 1.41 x10^-4^ |
| ILK Signaling | 3.71 x10^-4^ |
| Huntington's Disease Signaling | 4.07 x10^-4^ |
| Virus Entry via Endocytic Pathways | 5.5 x10^-4^ |
| Apoptosis Signaling | 5.5 x10^-4^ |
| Protein Kinase A Signaling | 5.89 x10^-4^ |
| Protein Ubiquitination Pathway | 7.08 x10^-4^ |
| Glycolysis I | 7.41 x10^-4^ |
| CDK5 Signaling | 9.12 x10^-4^ |
| NADH Repair | 0.001 |
| Putrescine Degradation III | 0.001 |
| Androgen Signaling | 0.001 |
| Regulation of Actin-based Motility by Rho | 0.002 |
| Aryl Hydrocarbon Receptor Signaling | 0.002 |
| Mitotic Roles of Polo-Like Kinase | 0.002 |
| Phospholipase C Signaling | 0.002 |
| Role of CHK Proteins in Cell Cycle Checkpoint Control | 0.003 |
| GÎ±q Signaling | 0.003 |
| PI3K/AKT Signaling | 0.003 |
| ERK/MAPK Signaling | 0.003 |
| Spermine and Spermidine Degradation I | 0.004 |
| Synaptic Long Term Potentiation | 0.005 |
| Caveolar-mediated Endocytosis Signaling | 0.005 |
| Integrin Signaling | 0.005 |
| fMLP Signaling in Neutrophils | 0.005 |
| RAR Activation | 0.006 |
| HIF1Î± Signaling | 0.006 |
| Pyrimidine Deoxyribonucleotides De Novo Biosynthesis I | 0.006 |
| IL-17A Signaling in Fibroblasts | 0.007 |
| Unfolded protein response | 0.007 |
| NRF2-mediated Oxidative Stress Response | 0.007 |
| Production of Nitric Oxide and Reactive Oxygen Species in Macrophages | 0.007 |
| Breast Cancer Regulation by Stathmin1 | 0.009 |
| 3-phosphoinositide Degradation | 0.009 |
| Cell Cycle: G2/M DNA Damage Checkpoint Regulation | 0.009 |
| Actin Nucleation by ARP-WASP Complex | 0.01 |
| Pyrimidine Ribonucleotides Interconversion | 0.01 |
| DNA Double-Strand Break Repair by Non-Homologous End Joining | 0.01 |
| Glycogen Degradation III | 0.01 |
| Guanine and Guanosine Salvage I | 0.01 |
| Adenine and Adenosine Salvage I | 0.01 |
| Glioma Invasiveness Signaling | 0.01 |
| Ethanol Degradation II | 0.011 |
| Tryptophan Degradation X (Mammalian, via Tryptamine) | 0.011 |
| Ethanol Degradation IV | 0.011 |
| GNRH Signaling | 0.011 |
| 14-3-3-mediated Signaling | 0.012 |
| FcÎ³ Receptor-mediated Phagocytosis in Macrophages and Monocytes | 0.012 |
| Antigen Presentation Pathway | 0.012 |
| LPS-stimulated MAPK Signaling | 0.013 |
| p70S6K Signaling | 0.013 |
| Pyrimidine Ribonucleotides De Novo Biosynthesis | 0.014 |
| ATM Signaling | 0.015 |
| Oxidative Ethanol Degradation III | 0.016 |
| Endoplasmic Reticulum Stress Pathway | 0.016 |
| Insulin Receptor Signaling | 0.016 |
| CXCR4 Signaling | 0.017 |
| RhoGDI Signaling | 0.017 |
| Noradrenaline and Adrenaline Degradation | 0.018 |
| nNOS Signaling in Neurons | 0.018 |
| Signaling by Rho Family GTPases | 0.018 |
| Tight Junction Signaling | 0.019 |
| PI3K Signaling in B Lymphocytes | 0.020 |
| IL-3 Signaling | 0.020 |
| D-myo-inositol-5-phosphate Metabolism | 0.022 |
| Role of Tissue Factor in Cancer | 0.023 |
| Superpathway of Inositol Phosphate Compounds | 0.023 |
| PPAR Signaling | 0.026 |
| Cholecystokinin/Gastrin-mediated Signaling | 0.027 |
| Xenobiotic Metabolism Signaling | 0.027 |
| Ceramide Biosynthesis | 0.027 |
| Glycogen Degradation II | 0.027 |
| Assembly of RNA Polymerase II Complex | 0.028 |
| Sonic Hedgehog Signaling | 0.029 |
| D-glucuronate Degradation I | 0.029 |
| Germ Cell-Sertoli Cell Junction Signaling | 0.029 |
| Hypoxia Signaling in the Cardiovascular System | 0.031 |
| Salvage Pathways of Pyrimidine Ribonucleotides | 0.031 |
| Hereditary Breast Cancer Signaling | 0.031 |
| UVB-Induced MAPK Signaling | 0.034 |
| Growth Hormone Signaling | 0.034 |
| Estrogen Receptor Signaling | 0.036 |
| Role of IL-17A in Psoriasis | 0.036 |
| B Cell Receptor Signaling | 0.037 |
| Axonal Guidance Signaling | 0.037 |
| Histamine Degradation | 0.038 |
| Fatty Acid Î²-oxidation I | 0.039 |
| Thrombin Signaling | 0.041 |
| tRNA Charging | 0.041 |
| RhoA Signaling | 0.042 |
| Phagosome Formation | 0.042 |
| Endothelin-1 Signaling | 0.042 |
| Molecular Mechanisms of Cancer | 0.042 |
| Neurotrophin/TRK Signaling | 0.043 |
| Agrin Interactions at Neuromuscular Junction | 0.046 |
| Retinol Biosynthesis | 0.047 |
| AMPK Signaling | 0.047 |
| The Visual Cycle | 0.047 |
